# Sleep-Deprivation Regulates α-2 Adrenergic Responses of Rat Hypocretin/Orexin Neurons

**DOI:** 10.1371/journal.pone.0016672

**Published:** 2011-02-08

**Authors:** Aaron Uschakov, Jeremy Grivel, Vesna Cvetkovic-Lopes, Laurence Bayer, Laurent Bernheim, Barbara E. Jones, Michel Mühlethaler, Mauro Serafin

**Affiliations:** 1 Département de Neurosciences fondamentales, Centre Médical Universitaire, Genève, Switzerland; 2 Department of Neurology and Neurosurgery, McGill University, Montreal Neurological Institute, Montreal, Quebec, Canada; The Research Center of Neurobiology-Neurophysiology of Marseille, France

## Abstract

We recently demonstrated, in rat brain slices, that the usual excitation by noradrenaline (NA) of hypocretin/orexin (hcrt/orx) neurons was changed to an inhibition following sleep deprivation (SD). Here we describe that in control condition (CC), i.e. following 2 hours of natural sleep in the morning, the α_2_-adrenergic receptor (α_2_-AR) agonist, clonidine, had no effect on hcrt/orx neurons, whereas following 2 hours of SD (SDC), it hyperpolarized the neurons by activating G-protein-gated inwardly rectifying potassium (GIRK) channels. Since concentrations of clonidine up to a thousand times (100 µM) higher than those effective in SDC (100 nM), were completely ineffective in CC, a change in the availability of G-proteins is unlikely to explain the difference between the two conditions. To test whether the absence of effect of clonidine in CC could be due to a down-regulation of GIRK channels, we applied baclofen, a GABA_B_ agonist known to also activate GIRK channels, and found that it hyperpolarized hcrt/orx neurons in that condition. Moreover, baclofen occluded the response to clonidine in SDC, indicating that absence of effect of clonidine in CC could not be attributed to down-regulation of GIRK channels. We finally tested whether α_2_-ARs were still available at the membrane in CC and found that clonidine could reduce calcium currents, indicating that α_2_-ARs associated with calcium channels remain available in that condition. Taken together, these results suggest that a pool of α_2_-ARs associated with GIRK channels is normally down-regulated (or desensitized) in hcrt/orx neurons to only become available for their inhibition following sleep deprivation.

## Introduction

The hypocretin/orexin (hcrt/orx) neurons of the hypothalamus play an important role in maintaining arousal, since their absence results in narcolepsy with cataplexy (for reviews, see [1]–[4]). The hcrt/orx neurons exert their influence during waking when they discharge selectively [5], [6], and through widespread projections [7] and excitatory actions upon multiple systems including all the activating and arousal systems in the brain [8].

Using rat brain slices, we have recently shown that hcrt/orx neurons are in turn normally excited by the multiple neurotransmitters of the arousal systems, including importantly noradrenaline [9]. However, using sleep-deprivation (SD), an approach used extensively to investigate sleep regulation and homeostasis (for recent review [10]), we discovered that following two hours of gentle SD, hcrt/orx neurons changed their response to noradrenaline from an excitation to an inhibition [11]. We proposed that such a phenomenon could contribute to the sleepiness that accompanies SD since during the natural sleep-wake cycle, the hcrt/orx neurons diminish firing before and cease firing during sleep [5], [6] and since their absence results in narcolepsy associated with increased daytime sleepiness (for review [12]).

Our goal in the present study was to investigate further the mechanisms underlying the emergence of inhibitory responses to noradrenaline following SD in rat brain slices. Our results suggest that a pool of α_2_-ARs associated with GIRK channels must become available for inhibition of hcrt/orx cells with SD.

## Results

### The α_2_ agonist clonidine hyperpolarizes hcrt/orx neurons following sleep-deprivation

**Figure 1:**
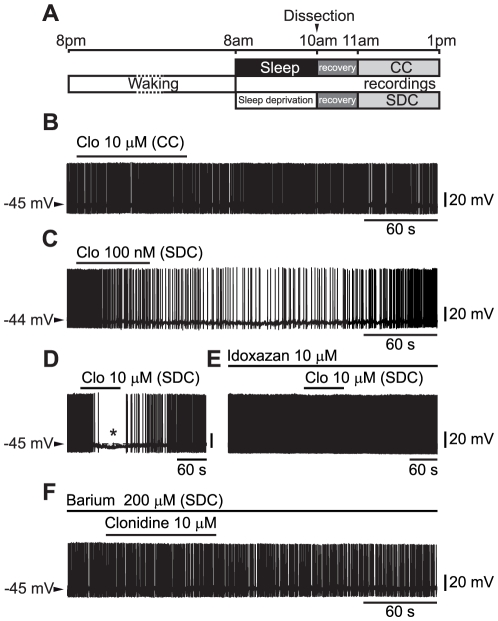
Effects of clonidine on identified hcrt/orx neurons. (A) Experimental protocol. Animals are maintained in a 12 h light/dark cycle (lights on from 8:00 am to 8:00 pm). After a normal cycle of sleep and waking, rats were either allowed to sleep from 8:00 to 10:00 am or kept fully awake; being gently sleep deprived for the same 2 hours interval. Recordings are made between 11 am and 1 pm in neurons obtained from rats which had slept (control condition, CC) or had been sleep deprived (sleep deprived condition, SDC) between 8 and 10 am. (B) Absence of effect of 10 µM clonidine in CC. (C–D) In SDC hcrt/orx neurons are inhibited by clonidine at both 100 nM (C) and 10 µM (D). (E) Idoxazan at 10 µM impedes the inhibitory and hyperpolarizing effect of clonidine in SDC. (F) Barium at 200 µM impedes the inhibitory and hyperpolarizing effect of clonidine in SDC.

To investigate the mechanism underlying the hyperpolarizing response to noradrenaline following sleep deprivation [11], hcrt/orx neurons were identified according to criteria illustrated in a previous publication [13] and tested for their response to the bath-application of the α_2_-AR agonist clonidine in either CC or SDC (see Fig. 1A). It was found that in CC, hcrt/orx neurons never responded to clonidine applied at either 100 nM (n = 6/6, not shown), 10 µM (n = 6/6, Fig. 1B) or even 100 µM (n = 3/3, not shown). In contrast, almost all hcrt/orx neurons decreased their activity in response to the agonist in SDC (n = 5/6 with clonidine at 100 nM, Fig. 1C and n = 9/10 with clonidine at 10 µM, Fig. 1D). These results indicate that the difference between the CC and SDC conditions cannot be explained by changing availability of G-proteins (see Discussion).

That the hyperpolarizing response was mediated by α_2_-ARs was confirmed by using a selective α_2_-AR antagonist, idoxazan (10 µM; n = 3/3, Fig. 1E), which in SDC completely blocked the response to clonidine at 10 µM. To test whether the inhibitory action of clonidine could result from the sleep deprivation procedure, we then applied the same procedure at a time of minimal homeostatic sleep pressure, i.e. during the dark period between 8 and 10 pm (see Methods), and found that, in this case, cells never responded to clonidine (n = 4/4, not shown).

These data show that the hyperpolarizing response of hcrt/orx neurons to noradrenaline that we have previously demonstrated in SDC [11] actually depend on α_2_-ARs which are regulated by SD. It is noteworthy that, in contrast, the depolarizing and excitatory responses to noradrenaline in CC described previously [11], were found here to depend on α_1_-ARs as they were mimicked by the α_1_-AR agonist L-phenylephrine. These α_1_-AR-dependent responses were however not regulated in the same manner as the α_2_-AR-dependent responses, as they did not change in between CC and SDC (n = 17/26 in CC and 5/12 in SDC; p = 0.15, Fischer exact test).

### Clonidine-dependent hyperpolarization following sleep-deprivation is mediated by GIRK channels

**Figure 2:**
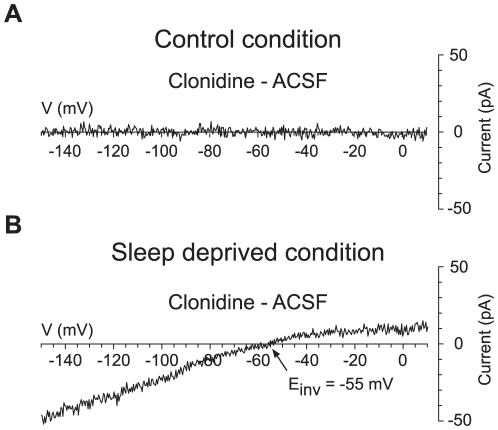
Effects of clonidine on hcrt/orx neurons in control and sleep deprived conditions. (A) In CC, as evident by subtraction of the current-voltage relationships obtained at voltages from −150 to +10 mV before (ACSF) and during application of clonidine at 10 µM, bath-application of clonidine did not elicit any current in hcrt/orx neurons. (B) In SDC, in contrast, subtraction of the currents measured in presence and absence of clonidine revealed a clonidine-dependent inward potassium current. Indeed, the inversion potential is around −55 mV which is the inversion potential of potassium in the experimental conditions (see Methods).

Since the activation of α_2_-ARs is known to hyperpolarize neurons through the opening of GIRK channels [14], we tested whether such channels were indeed implicated in the present effects of clonidine. For that purpose we first used barium which, at a small concentration (200 µM), is known to act as a blocker of GIRK channels [15]. When clonidine at 10 µM was applied in presence of barium in SDC, it was found to have no effect (n = 4/4 in current clamp, Fig. 1F, and n = 3/3 in voltage-clamp, not shown). To further demonstrate the implication of GIRK channels in SDC, clonidine effects were studied while applying voltage ramps from −150 to +10 mV. When, in CC, current-voltage relationships before (artificial cerebrospinal fluid, ACSF, see Methods) and during application of clonidine at 10 µM were compared, we found that clonidine had no effect (Fig. 2A, ACSF vs clonidine, n = 5). In contrast, in SDC, current-voltage relationships before (ACSF) and during application of 10 µM clonidine revealed currents (Fig. 2B, n = 4; mean E_inv_ ± SEM = −54.0±2.94 mV) that were small and outward at potentials positive to E_K_ (set at −55 mV, see Methods) but strongly rectified and became inward below E_K_.

### GIRK channels are not down-regulated in the control condition

**Figure 3:**
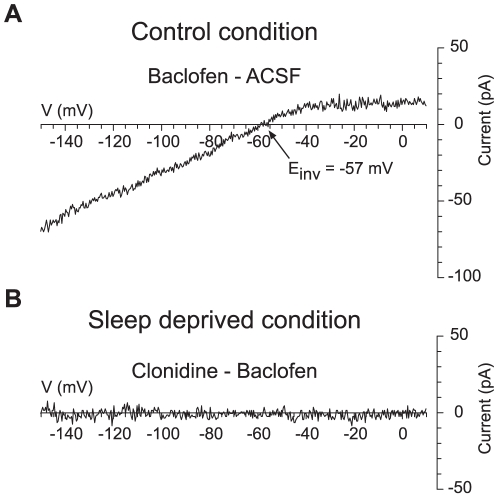
GIRK channels are present on hcrt/orx neurons. (A) Subtraction of the current-voltage relationships obtained at voltages from −150 to +10 mV before (ACSF) and during application of baclofen at 10 µM. revealed a baclofen-dependent inward current that reversed around −55 mV, which is the inversion potential of potassium in the experimental conditions. (B) In SDC, subtraction of the current-voltage relationships from −150 to +10 mV measured successively in presence of baclofen alone (baclofen) and following subsequent addition of clonidine at 10 µM demonstrated that baclofen occluded the effect of clonidine.

One possible explanation for the above results could be that GIRK channels are not available in CC and become available in SDC. To test this hypothesis, we applied baclofen, a selective GABA_B_ receptor agonist, also known to activate GIRK channels in many CNS neurons [15]. In current-clamp, whereas clonidine had no effect in CC, baclofen at 10 µM, hyperpolarized every cell (n = 5/5, not shown). To demonstrate that the baclofen effect was due to the presence of GIRK channels, we next studied the effect of baclofen using voltage-clamp ramps. As illustrated in Fig. 3A, baclofen triggered an inwardly rectifying current (n = 4), thereby suggesting that in CC, GIRK channels are still available on the membrane.

To exclude the possibility that clonidine and baclofen acted on different populations of GIRK channels, we applied clonidine in SDC, following a prior application of baclofen, and found that clonidine evoked no additional effect in that condition (Fig. 3B, n = 4; compare with Fig. 2B). Altogether these data indicate that the lack of effect of clonidine in CC cannot be explained by a down-regulation of GIRK channels.

### Clonidine reduces calcium currents in control condition

Another possibility to explain the lack of responsiveness of hcrt/orx neurons to clonidine in CC could be that the α_2_-ARs become down-regulated (or desensitized) in that situation [14], [16]. If this were the case, one would expect that modulation of other membrane currents by these receptors would also be impeded.

**Figure 4:**
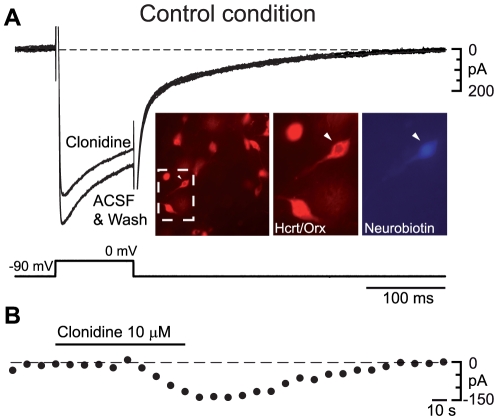
α_2_-ARs activation in the control condition reduces total calcium current in identified hcrt/orx neurons. (A) Total calcium current measured in response to a voltage step from −90 to 0 mV in a typical hcrt/orx neuron before (control), during (clonidine) and after (wash) bath-application of clonidine at 10 µM. In the insets, the recorded neuron (arrowhead) is shown to be Hcrt/Orx-positive (middle inset) and neurobiotin-positive (right inset). (B) Time-dependent reduction of the total calcium current following transient application of clonidine at 10 µM. Experiments were done in presence of 10 µM NBQX, 50 µM D-APV, 30 µM bicuculline, 1 µM tetrodotoxin, 20 mM TEA, 4 mM 4-AP and 3 mM ClCs in the ACSF.

Since activation of α_2_-ARs often produces a decrease in calcium currents [17], we examined whether this response to clonidine occurred in CC. For this purpose, cells had first to be identified electrophysiologically [13] using a regular electrode solution and then patched a second time with a cesium-containing electrode to decrease potassium currents. Some cells were in addition injected with neurobiotin for immunohistochemical identification (inset of Fig. 4A). Electrophysiologically identified hcrt/orx cells were then submitted, every 10 seconds, to a voltage step from −90 to 0 mV to evoke an inward calcium current (Fig. 4A). As evidenced in Fig. 4A and B, application of clonidine at 10 µM reversibly decreased the total calcium current (n = 5). These results suggest that in CC, functional α_2_-ARs linked to calcium channels must still be available at the membrane.

## Discussion

This study shows that in CC, hcrt/orx neurons were insensitive to the α_2_-AR agonist, clonidine, whereas they were hyperpolarized, through the activation of GIRK channels, in SDC. Several lines of evidence suggest that a pool of α_2_-ARs associated with GIRK channels becomes available for inhibition of hcrt/orx cells with sleep deprivation.

In our original study on the consequences of SD [11], we showed that, in juvenile rat brain slices, noradrenaline usually depolarized and excited hcrt/orx neurons in CC, but hyperpolarized and inhibited the same cells in SDC. These data stood in contrast to those in mice, in which noradrenaline always hyperpolarized hcrt/orx cells [18], [19]. It is noteworthy that since our own (unpublished) observations are in agreement with these data, the mouse model could not be used in the present study to further explore this process. In mice, the hyperpolarization of hcrt/orx cells depended on α_2_-ARs, but α_1_-dependent excitatory responses could also be evoked by L-phenylephrine, although only after the selective blockage by idoxazan [18], indicating that under baseline laboratory conditions in mice the α_2_-ARs predominate. Not surprisingly, we found that the hyperpolarizing responses to noradrenaline in SDC depended on α_2_-ARs since they could be mimicked by clonidine and antagonized by idoxazan. It is noteworthy, in contrast, that the excitatory action of noradrenaline observed in CC, which depends on α_1_-ARs, since it was mimicked by L-phenylephrine, was not similarly regulated, since it did not change significantly between CC and SDC. This indicates that the inhibitory effect of noradrenaline, apparent in SDC [11], results from the emergence of an α_2_-AR dependent response which overrides the α_1_-AR dependent one. The reasons for the species differences in baseline responses to noradrenaline on the one hand and in responses to SD on the other hand, with hyperpolarizations as shown here in rats, versus depolarisations (following potentiation of glutamate responses) in mice [20] are not understood. Functionally however, with respect to the sleepiness normally accompanying SD (see final paragraph of Discussion), it must be stressed that the rat model seems more relevant than the mouse one.

This inhibitory action of clonidine in SDC is due to the opening of GIRK channels [15], [21] as indicated by the voltage-dependence of the current elicited by the agonist with its characteristically marked inward rectification at potentials negative to E_K_ and its sensitivity to minute amounts of barium. These results are congruent with a number of studies in many different systems indicating that inhibitory actions of α_2_-AR activation can be mediated by activation of GIRK channels [14].

As clonidine was completely ineffective in CC, it appears that the regulation of noradrenergic responses in rat hcrt/orx cells is entirely dependent on the presence (in SDC) or absence (in CC) of α_2_-AR-dependent responses. To explain the absence of response in CC, several hypotheses can be evoked (for reviews [22]–[24]): a down-regulation of GIRK channels [24], a change in the availability of G-proteins (mediated by regulators of G-proteins, RGS [25] or guanine-nucleotide-dissociation inhibitor proteins, GDI [26]), a general desensitization of α_2_-ARs by clonidine [14], [24], and finally a down–regulation (or heterologus desensitization) of a pool of α_2_-ARs associated with the GIRK channels [14], [16], [24].

With respect to the first hypothesis, we were able to demonstrate that down-regulation of GIRK channels could not be involved in the absence of response to clonidine in CC since GIRK-dependent responses to baclofen could still be evoked in that condition. In addition, occlusion experiments in SDC between clonidine and baclofen showed that the population of GIRK channels responding to both agonists must be the same. The second hypothesis, that a change in the availability of G-proteins under the regulatory influence of RGS or GDI proteins could explain the switch between CC and SDC, is not supported by our results either. Indeed, in studies that have tackled the question of modulation of responses to agonists by the availability of G-proteins, data suggest modest shifts in dose-responses [27]–[29]. In our experiments however, even at concentrations that were one thousand times higher in CC (100 µM) than those efficient in SDC (100 nM), clonidine was completely ineffective. The third hypothesis, that the α_2_-ARs are desensitized in CC, is certainly not true for all α_2_-ARs, since clonidine was still able in that condition to decrease the total calcium current, as is the case in other preparations [17]. In addition, for those receptors responsible for the hyperpolarization in SDC, the mechanism is certainly not a homologous desensitization involving the continuous presence of the agonist [24]. Indeed, noradrenaline release during the two conditions must be exactly opposite to what would be needed for such an agonist-mediated desensitization to occur. In CC, the animals have slept for 2 hours and thus noradrenaline release must have been relatively low, whereas during the waking associated with SD, noradrenaline release must certainly have been higher [30], [31]. It is nonetheless during SD that the α_2_-dependent responses develop.

At this stage, with no evidence for either a down-regulation of GIRK channels or a change in G-protein availability, together with evidence for a persisting action of clonidine on calcium currents in CC, one is left with the hypothesis that, those α_2_-ARs associated with calcium channels and those associated with GIRK channels must be differentially regulated. Those receptors associated with GIRK channels could thus form a pool that is down-regulated (or heterologously desensitized) in CC and which is up-regulated (or resensitized) selectively with sleep-deprivation to provide inhibition of hcrt/orx neurons. The signal associated with the sleep deprivation process that triggers that change remains to be investigated.

Functionally, the present data suggest that changing the response of hcrt/orx neurons to noradrenaline through the emergence of functional α_2_-ARs during sleep deprivation could contribute to the increased sleepiness associated with that condition. Regulation of adrenergic receptors could represent a homeostatic response to the continuous discharge by the hcrt/orx neurons that would occur during enforced waking [6]. Increasing hyperpolarization of the hcrt/orx neurons through the α_2_-ARs would serve to dampen their responsiveness and decrease their firing such as to indirectly facilitate sleep by removing their excitatory influence from their diverse targets, including the other arousal systems of the brain.

## Materials and Methods

### Experimental conditions

Young (17–21 days) Sprague-Dawley rats (Laboratoires Charles River, France) were maintained in a 12 h light/dark cycle (lights on from 8:00 am to 8:00 pm) and treated according to the regulations of the Swiss Federal Veterinary Office. The study was reviewed and approved by the “office vétérinaire cantonal” (***approval ID: 31.1.1007/3248/0***). As previously described, after a normal cycle of sleep and waking, rats were either allowed to sleep for 2 h from 8:00 to 10:00 am or kept fully awake; being gently sleep deprived (for details see [11], [32] for the same 2 hours interval. Then, neurons were recorded between 11 am and 1 pm in control condition (CC) and sleep deprived condition (SDC), respectively. Some experiments were also conducted at night (between 8 and 10 pm), in which case night-vision goggles were used.

### Slice preparation

250 µm-thick hypothalamic coronal slices containing the hcrt/orx neurons were obtained at 10 am from CC or SD rats. Slices were maintained *in vitro* for only 3 hours following sacrifice since EEG studies and our own previous study demonstrated that the consequences of sleep deprivation can be observed during the three hours following the deprivation protocol [11], [33]. Tissue was left to recover for 1 hour at room temperature in artificial cerebrospinal fluid (ACSF) containing (in mM): 130 NaCl, 5 KCl, 1.25 KH_2_PO_4_, 1.3 MgSO_4_, 20 NaHCO_3_, 10 glucose and 2.4 CaCl_2_, and bubbled with 95% O_2_ and 5% CO_2_. Subsequently, individual slices were transferred to a thermoregulated (32°C) recording chamber mounted on an upright microscope (Axioskop; Zeiss, Oberkochen, Germany) equipped with an infrared camera and continuously superfused at 4–5 ml/min with ACSF.

### Electrophysiology

Patch pipettes (4 to 7 MΩ) were pulled on a DMZ universal puller (Zeitz-Instrumente, Munich, Germany) from borosilicate glass capillaries (GC150F-10; Harvard Apparatus, France) and filled with a solution containing (in mM) 126 KMeSO_4_, 4 KCl, 5 MgCl_2_, 0.1 BAPTA, 10 HEPES, 8 phosphocreatine, 3 ATP, 0.1 GTP (pH 7.3, 285–300 mOsm) and 2% neurobiotin (Vector Labs, REACTOLAB, S.A., Switzerland) to allow subsequent immunohistochemical identification as previously described [13]. Whole-cell recordings were performed between 11 am and 1 pm in the current-clamp or voltage-clamp mode using Axopatch 200A or 200B amplifiers (Molecular devices, Sunnywale, CA, USA). The membrane potential values were not compensated for junction potential (estimated at −9.6 mV). Voltage-clamp data were accepted when series resistance was <20 MΩ and changed <15% during recording, and analysed with Clampfit 10.0 (Molecular devices).

In voltage-clamp experiments designed to reveal a baclofen or clonidine-dependent activation of GIRK currents, the external potassium concentration was changed from 6.25 to 16 mM to raise the driving force for this ion. In these experimental conditions the equilibrium potential for potassium was estimated from the Nernst equation as E_K_  =  RT/F*ln([K^+^]_out_/[K^+^]_in_)  =  −55 mV. 100 ms-long ramps from −150 to +10 mV were repeated every 10 seconds before and following addition of clonidine or baclofen, respectively. Experiments were done in presence of 1 µM tetrodotoxin (TTX, Latoxan, Rosans, France), 10 µM NBQX, 50 µM D-APV, 30 µM bicuculline (all from Tocris) to block sodium action potentials and synaptic activity. Current traces are signals averaged from three traces for ACSF, baclofen and clonidine, respectively.

In voltage-clamp experiments designed to investigate a clonidine-dependent effect on the total calcium current, the cells were first identified electrophysiologically with the above intrapipette solution. Then, after 20 minutes, the same electrophysiologically identified hcrt/orx cell was re-patched with a cesium-containing intrapipette solution (in mM): CsMeSulfonate 140, CsCl 10, Hepes 10, MgCl_2_ 4, BAPTA 0.1, phosphocreatine 8, Na_2_-ATP 2, Na_2_-GTP 0.4 and neurobiotin 2% (pH = 7.4, 290–310 mOsm) whereby blocking potassium currents allowed the isolation of the total calcium current. Experiments were done in presence of 1 µM TTX to block sodium channels; 20 mM TEA, 4 mM 4-AP, 3 mM ClCs (all from Sigma) to block potassium channels externally and 10 µM NBQX, 50 µM D-APV, 30 µM bicuculline (all from Tocris) to block synaptic activity. A 100 ms-long voltage step from −90 to 0 mV was repeated every 10 seconds allowing total calcium current to be recorded before, during and following transient application of clonidine (Sigma).
